# Bis(μ-2-methyl-8-oxidoquinolin-1-ium-κ^2^ *O*:*O*)bis­[(acetato-κ^2^ *O*,*O*′)(2-methyl-8-oxidoquinolin-1-ium-κ*O*)bis­(nitrato-κ^2^ *O*,*O*′)lanthanum(III)]

**DOI:** 10.1107/S1600536809055743

**Published:** 2010-01-16

**Authors:** Yousef Fazaeli, Mostafa M. Amini, Seik Weng Ng

**Affiliations:** a Department of Chemistry, General Campus, Shahid Beheshti University, Tehran 1983963113, Iran; b Department of Chemistry, University of Malaya, 50603 Kuala Lumpur, Malaysia

## Abstract

The *N*-heterocycles in the centrosymmetric title compound, [La_2_(C_10_H_9_NO)_4_(CH_3_COO)_2_(NO_3_)_4_], exist in the zwitterionic form. One heterocycle binds to a metal center whereas the other bridges two metal centers. Each La atom is chelated by an acetate and two nitrate groups and is surrounded by nine O atoms in a distorted tricapped trigonal-prismatic coordination environment. The N—H groups form intra­molecular N—H⋯O hydrogen bonds. One of the nitrate ions is disordered over two positions in a 0.80 (3):0.20 (3) occupancy ratio.

## Related literature

For bis­(μ-2-methyl­quinolin-1-ium-8-olato)bis­[(2-methyl­quin­o­lin-1-ium-8-olato-)tris­(nitrato)lanthanum(III)], see: Faza­eli *et al.* (2009 ▶).
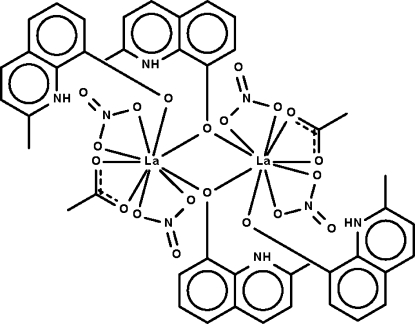


## Experimental

### 

#### Crystal data

[La_2_(C_10_H_9_NO)_4_(C_2_H_3_O_2_)_2_(NO_3_)_4_]
*M* *_r_* = 1280.68
Monoclinic, 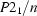

*a* = 11.3686 (8) Å
*b* = 17.5807 (12) Å
*c* = 13.0265 (10) Å
β = 104.820 (1)°
*V* = 2517.0 (3) Å^3^
*Z* = 2
Mo *K*α radiation
μ = 1.76 mm^−1^
*T* = 295 K
0.35 × 0.15 × 0.05 mm

#### Data collection

Bruker SMART APEX diffractometer
Absorption correction: multi-scan (*SADABS*; Sheldrick, 1996 ▶) *T*_min_ = 0.578, *T*_max_ = 0.917
15685 measured reflections
5769 independent reflections
4544 reflections with *I* > 2σ(*I*)
*R*_int_ = 0.030

#### Refinement

*R*[*F* ^2^ > 2σ(*F* ^2^)] = 0.032
*wR*(*F* ^2^) = 0.112
*S* = 1.11
5769 reflections
358 parameters
58 restraints
H atoms treated by a mixture of independent and constrained refinement
Δρ_max_ = 0.84 e Å^−3^
Δρ_min_ = −0.77 e Å^−3^

### 

Data collection: *APEX2* (Bruker, 2008 ▶); cell refinement: *SAINT* (Bruker, 2008 ▶); data reduction: *SAINT*; program(s) used to solve structure: *SHELXS97* (Sheldrick, 2008 ▶); program(s) used to refine structure: *SHELXL97* (Sheldrick, 2008 ▶); molecular graphics: *X-SEED* (Barbour, 2001 ▶); software used to prepare material for publication: *publCIF* (Westrip, 2010 ▶).

## Supplementary Material

Crystal structure: contains datablocks global, I. DOI: 10.1107/S1600536809055743/bt5149sup1.cif

Structure factors: contains datablocks I. DOI: 10.1107/S1600536809055743/bt5149Isup2.hkl

Additional supplementary materials:  crystallographic information; 3D view; checkCIF report

## Figures and Tables

**Table 1:** Hydrogen-bond geometry (Å, °)

*D*—H⋯*A*	*D*—H	H⋯*A*	*D*⋯*A*	*D*—H⋯*A*
N1—H1⋯O3	0.86 (1)	2.06 (1)	2.910 (5)	173 (5)
N2—H2⋯O4	0.86 (1)	2.20 (3)	2.926 (5)	143 (5)

**Fig. 1.**
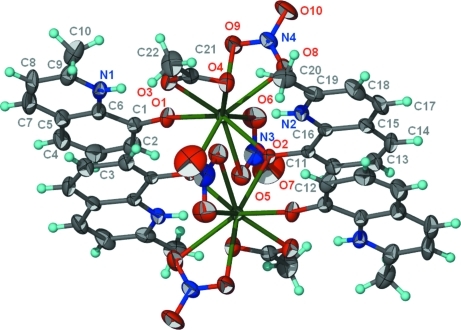
Anisotropic displacement ellipsoid plot (Barbour, 2001) of the title compound; ellipsoids are drawn at the 50% probability level and H atoms of arbitrary radius. The minor occupied site of the disorder nitrate ion is not shown.

## supplementary crystallographic information

### Experimental

2-Methyl-8-hydroxyquinoline (0.16 g, 1 mmol) and sodium acetate (0.08, 1 mmol) were added reacted with lanthanum nitrate hexahydrate (0.43 g, 1 mmol) in methanol (10 ml). The mixture was stirred two hours. Slow evaporation of the solution gave deep orange color crystals that are stable when heated up to 573 K.

### Refinement

Carbon-bound H-atoms were placed in calculated positions (C–H 0.93–0.96 Å) and were included in the refinement in the riding model approximation, with *U*(H) set to 1.2–1.5*U*(C).

The nitrogen-bound H atoms were located in a difference Fourier map, and were refined with a distance restraint of N–H 0.86±0.01 Å; the displacement parameters of these H atoms were refined.

One of the nitrate ions is disordered over two positions; the occupancy of the major occupied site refined to 0.80 (3). The N–O distances as well as the O···O distances were restrained to be equal within 0.01 Å of each other. The four-atom unit was restrained to be  planar. The isotropic displacement parameters  of the primed atoms were set to the equivalent isotropic temperature factors of the unprimed ones, and the anisotropic temperature factors of these unprimed atoms were restrained to be nearly isotropic.

### Crystal data

**Table:**

[La_2_(C_10_H_9_NO)_4_(C_2_H_3_O_2_)_2_(NO_3_)_4_]	*F*(000) = 1272
*M**_r_* = 1280.68	*D*_x_ = 1.690 Mg m^−^^3^
Monoclinic, *P*2_1_/*n*	Mo *K*α radiation, λ = 0.71073 Å
Hall symbol: -P 2yn	Cell parameters from 7894 reflections
*a* = 11.3686 (8) Å	θ = 2.2–27.8°
*b* = 17.5807 (12) Å	µ = 1.76 mm^−^^1^
*c* = 13.0265 (10) Å	*T* = 295 K
β = 104.820 (1)°	Block, orange
*V* = 2517.0 (3) Å^3^	0.35 × 0.15 × 0.05 mm
*Z* = 2	

### Data collection

**Table:**

Bruker SMART APEX diffractometer	5769 independent reflections
Radiation source: fine-focus sealed tube	4544 reflections with *I* > 2σ(*I*)
graphite	*R*_int_ = 0.030
ω scans	θ_max_ = 27.5°, θ_min_ = 2.0°
Absorption correction: multi-scan (*SADABS*; Sheldrick, 1996)	*h* = −14→12
*T*_min_ = 0.578, *T*_max_ = 0.917	*k* = −17→22
15685 measured reflections	*l* = −16→16

### Refinement

**Table:**

Refinement on *F*^2^	Primary atom site location: structure-invariant direct methods
Least-squares matrix: full	Secondary atom site location: difference Fourier map
*R*[*F*^2^ > 2σ(*F*^2^)] = 0.032	Hydrogen site location: inferred from neighbouring sites
*wR*(*F*^2^) = 0.112	H atoms treated by a mixture of independent and constrained refinement
*S* = 1.11	*w* = 1/[σ^2^(*F*_o_^2^) + (0.0493*P*)^2^ + 4.7409*P*] where *P* = (*F*_o_^2^ + 2*F*_c_^2^)/3
5769 reflections	(Δ/σ)_max_ = 0.001
358 parameters	Δρ_max_ = 0.84 e Å^−^^3^
58 restraints	Δρ_min_ = −0.76 e Å^−^^3^

### Special details

**Table:**

Geometry. All e.s.d.'s (except the e.s.d. in the dihedral angle between two l.s. planes) are estimated using the full covariance matrix. The cell e.s.d.'s are taken into account individually in the estimation of e.s.d.'s in distances, angles and torsion angles; correlations between e.s.d.'s in cell parameters are only used when they are defined by crystal symmetry. An approximate (isotropic) treatment of cell e.s.d.'s is used for estimating e.s.d.'s involving l.s. planes.

### Fractional atomic coordinates and isotropic or equivalent isotropic displacement parameters (Å^2^)

**Table:**

	*x*	*y*	*z*	*U*_iso_*/*U*_eq_	Occ. (<1)
La1	0.59038 (2)	0.543193 (14)	0.648779 (18)	0.02973 (9)	
O1	0.4836 (3)	0.53330 (19)	0.7782 (3)	0.0433 (8)	
O2	0.5705 (3)	0.54741 (16)	0.4544 (2)	0.0298 (6)	
O3	0.6618 (3)	0.4169 (2)	0.7427 (3)	0.0490 (9)	
O4	0.7505 (3)	0.44741 (19)	0.6178 (3)	0.0466 (9)	
O5	0.3958 (7)	0.6311 (4)	0.5777 (6)	0.0566 (18)	0.80 (3)
O5'	0.420 (3)	0.6466 (19)	0.570 (2)	0.057*	0.20 (3)
O6	0.5573 (5)	0.6913 (4)	0.6548 (12)	0.085 (3)	0.80 (3)
O6'	0.567 (2)	0.677 (2)	0.704 (3)	0.085*	0.20 (3)
O7	0.3882 (10)	0.7522 (4)	0.6030 (10)	0.103 (3)	0.80 (3)
O7'	0.428 (4)	0.7585 (17)	0.638 (4)	0.103*	0.20 (3)
O8	0.7936 (4)	0.6159 (2)	0.6401 (3)	0.0556 (10)	
O9	0.7741 (4)	0.5927 (3)	0.7967 (3)	0.0620 (11)	
O10	0.9344 (4)	0.6514 (3)	0.7766 (4)	0.0782 (14)	
N1	0.5090 (4)	0.4012 (2)	0.8897 (3)	0.0398 (9)	
N2	0.7810 (3)	0.4997 (2)	0.4128 (3)	0.0360 (9)	
N3	0.4457 (7)	0.6920 (3)	0.6115 (6)	0.0563 (18)	0.80 (3)
N3'	0.472 (3)	0.6941 (14)	0.638 (2)	0.056*	0.20 (3)
N4	0.8361 (4)	0.6214 (3)	0.7390 (4)	0.0470 (10)	
C1	0.4074 (4)	0.5195 (3)	0.8361 (3)	0.0363 (10)	
C2	0.3143 (5)	0.5680 (3)	0.8457 (4)	0.0474 (12)	
H2A	0.3038	0.6140	0.8093	0.057*	
C3	0.2355 (5)	0.5488 (4)	0.9092 (4)	0.0597 (16)	
H3	0.1734	0.5822	0.9129	0.072*	
C4	0.2472 (6)	0.4829 (4)	0.9653 (5)	0.0618 (16)	
H4	0.1944	0.4721	1.0074	0.074*	
C5	0.3394 (5)	0.4310 (3)	0.9597 (4)	0.0494 (13)	
C6	0.4183 (5)	0.4505 (3)	0.8952 (4)	0.0384 (11)	
C7	0.3617 (6)	0.3623 (4)	1.0154 (4)	0.0602 (16)	
H7	0.3124	0.3482	1.0593	0.072*	
C8	0.4531 (7)	0.3161 (3)	1.0068 (4)	0.0597 (17)	
H8	0.4652	0.2703	1.0439	0.072*	
C9	0.5307 (6)	0.3360 (3)	0.9425 (4)	0.0503 (13)	
C10	0.6331 (7)	0.2879 (4)	0.9293 (5)	0.0681 (18)	
H10A	0.6972	0.3198	0.9180	0.102*	
H10B	0.6053	0.2549	0.8692	0.102*	
H10C	0.6632	0.2579	0.9920	0.102*	
C11	0.6280 (4)	0.5961 (3)	0.4055 (3)	0.0302 (9)	
C12	0.5900 (4)	0.6680 (3)	0.3746 (4)	0.0396 (11)	
H12	0.5207	0.6872	0.3908	0.048*	
C13	0.6541 (5)	0.7138 (3)	0.3183 (4)	0.0509 (13)	
H13	0.6259	0.7628	0.2994	0.061*	
C14	0.7547 (5)	0.6897 (3)	0.2905 (4)	0.0512 (13)	
H14	0.7932	0.7208	0.2513	0.061*	
C15	0.7998 (4)	0.6162 (3)	0.3222 (4)	0.0410 (11)	
C16	0.7377 (4)	0.5703 (3)	0.3809 (3)	0.0326 (9)	
C17	0.9041 (5)	0.5839 (4)	0.2988 (5)	0.0527 (14)	
H17	0.9478	0.6121	0.2607	0.063*	
C18	0.9410 (5)	0.5128 (4)	0.3310 (5)	0.0586 (16)	
H18	1.0094	0.4927	0.3141	0.070*	
C19	0.8779 (4)	0.4684 (3)	0.3897 (4)	0.0443 (12)	
C20	0.9120 (5)	0.3891 (3)	0.4264 (5)	0.0587 (15)	
H20A	0.8942	0.3810	0.4937	0.088*	
H20B	0.9974	0.3816	0.4337	0.088*	
H20C	0.8664	0.3537	0.3754	0.088*	
C21	0.7278 (4)	0.3996 (3)	0.6821 (4)	0.0436 (11)	
C22	0.7752 (6)	0.3200 (4)	0.6835 (6)	0.074 (2)	
H22A	0.8254	0.3085	0.7529	0.110*	
H22B	0.8225	0.3155	0.6323	0.110*	
H22C	0.7082	0.2851	0.6659	0.110*	
H1	0.552 (4)	0.410 (3)	0.845 (3)	0.043 (14)*	
H2	0.748 (4)	0.472 (2)	0.452 (3)	0.042 (15)*	

### Atomic displacement parameters (Å^2^)

**Table:**

	*U*^11^	*U*^22^	*U*^33^	*U*^12^	*U*^13^	*U*^23^
La1	0.03124 (15)	0.03346 (15)	0.02601 (13)	−0.00429 (11)	0.01007 (9)	−0.00058 (10)
O1	0.055 (2)	0.046 (2)	0.0370 (17)	−0.0002 (16)	0.0253 (16)	0.0023 (14)
O2	0.0299 (15)	0.0332 (16)	0.0284 (14)	−0.0021 (12)	0.0112 (12)	0.0018 (12)
O3	0.053 (2)	0.051 (2)	0.047 (2)	0.0087 (18)	0.0209 (17)	0.0113 (17)
O4	0.050 (2)	0.041 (2)	0.054 (2)	0.0048 (16)	0.0236 (17)	0.0087 (16)
O5	0.052 (3)	0.039 (3)	0.076 (3)	−0.006 (2)	0.011 (3)	−0.003 (3)
O6	0.073 (4)	0.033 (3)	0.127 (7)	−0.005 (3)	−0.013 (4)	−0.011 (3)
O7	0.116 (6)	0.073 (4)	0.119 (6)	0.053 (4)	0.026 (5)	−0.015 (4)
O8	0.061 (2)	0.065 (3)	0.044 (2)	−0.026 (2)	0.0209 (18)	−0.0052 (18)
O9	0.055 (2)	0.093 (3)	0.0380 (19)	−0.027 (2)	0.0114 (17)	0.002 (2)
O10	0.045 (2)	0.105 (4)	0.081 (3)	−0.033 (2)	0.010 (2)	−0.011 (3)
N1	0.053 (3)	0.039 (2)	0.0288 (19)	−0.0081 (19)	0.0135 (18)	−0.0017 (16)
N2	0.034 (2)	0.045 (2)	0.0330 (19)	−0.0017 (18)	0.0151 (16)	−0.0014 (17)
N3	0.062 (4)	0.055 (4)	0.053 (4)	0.019 (3)	0.019 (3)	−0.006 (3)
N4	0.039 (2)	0.051 (3)	0.050 (2)	−0.011 (2)	0.0106 (19)	−0.008 (2)
C1	0.043 (3)	0.041 (3)	0.026 (2)	−0.008 (2)	0.0112 (19)	−0.0061 (18)
C2	0.057 (3)	0.052 (3)	0.035 (2)	0.001 (3)	0.016 (2)	−0.002 (2)
C3	0.043 (3)	0.096 (5)	0.043 (3)	0.000 (3)	0.017 (2)	−0.010 (3)
C4	0.059 (4)	0.089 (5)	0.047 (3)	−0.020 (3)	0.030 (3)	−0.006 (3)
C5	0.054 (3)	0.065 (4)	0.031 (2)	−0.021 (3)	0.014 (2)	−0.006 (2)
C6	0.048 (3)	0.042 (3)	0.026 (2)	−0.013 (2)	0.0116 (19)	−0.0040 (19)
C7	0.083 (4)	0.064 (4)	0.042 (3)	−0.025 (3)	0.032 (3)	−0.002 (3)
C8	0.104 (5)	0.035 (3)	0.044 (3)	−0.033 (3)	0.026 (3)	−0.002 (2)
C9	0.077 (4)	0.039 (3)	0.035 (2)	−0.009 (3)	0.014 (2)	−0.002 (2)
C10	0.101 (5)	0.054 (4)	0.050 (3)	0.007 (4)	0.021 (3)	0.008 (3)
C11	0.027 (2)	0.039 (2)	0.0233 (18)	−0.0047 (18)	0.0037 (16)	0.0001 (17)
C12	0.038 (3)	0.038 (3)	0.044 (3)	0.000 (2)	0.012 (2)	0.002 (2)
C13	0.052 (3)	0.047 (3)	0.052 (3)	−0.011 (3)	0.010 (2)	0.003 (2)
C14	0.060 (3)	0.053 (3)	0.043 (3)	−0.014 (3)	0.017 (2)	0.013 (2)
C15	0.032 (2)	0.061 (3)	0.030 (2)	−0.014 (2)	0.0090 (18)	−0.006 (2)
C16	0.029 (2)	0.043 (3)	0.0243 (19)	−0.0067 (19)	0.0034 (16)	−0.0024 (18)
C17	0.045 (3)	0.065 (4)	0.054 (3)	−0.019 (3)	0.023 (2)	−0.007 (3)
C18	0.039 (3)	0.079 (4)	0.069 (4)	−0.007 (3)	0.035 (3)	−0.020 (3)
C19	0.033 (2)	0.057 (3)	0.042 (3)	0.001 (2)	0.008 (2)	−0.013 (2)
C20	0.050 (3)	0.064 (4)	0.062 (4)	0.012 (3)	0.013 (3)	−0.010 (3)
C21	0.035 (3)	0.045 (3)	0.049 (3)	0.004 (2)	0.008 (2)	0.011 (2)
C22	0.075 (4)	0.049 (4)	0.105 (6)	0.025 (3)	0.038 (4)	0.019 (4)

### Geometric parameters (Å, °)

**Table:**

La1—O1	2.322 (3)	C3—C4	1.358 (9)
La1—O2	2.485 (3)	C3—H3	0.9300
La1—O6'	2.49 (3)	C4—C5	1.405 (9)
La1—O2^i^	2.536 (3)	C4—H4	0.9300
La1—O3	2.564 (4)	C5—C7	1.399 (8)
La1—O4	2.586 (3)	C5—C6	1.418 (7)
La1—O9	2.603 (4)	C7—C8	1.346 (9)
La1—O5'	2.66 (3)	C7—H7	0.9300
La1—O6	2.635 (6)	C8—C9	1.408 (8)
La1—O5	2.663 (7)	C8—H8	0.9300
La1—O8	2.668 (4)	C9—C10	1.484 (9)
La1—C21	2.942 (5)	C10—H10A	0.9600
O1—C1	1.309 (5)	C10—H10B	0.9600
O2—C11	1.334 (5)	C10—H10C	0.9600
O2—La1^i^	2.536 (3)	C11—C12	1.363 (6)
O3—C21	1.257 (6)	C11—C16	1.438 (6)
O4—C21	1.260 (6)	C12—C13	1.410 (7)
O5—N3	1.239 (6)	C12—H12	0.9300
O5'—N3'	1.241 (7)	C13—C14	1.353 (8)
O6—N3	1.250 (6)	C13—H13	0.9300
O6'—N3'	1.241 (7)	C14—C15	1.412 (8)
O7—N3	1.233 (5)	C14—H14	0.9300
O7'—N3'	1.240 (7)	C15—C17	1.416 (7)
O8—N4	1.258 (6)	C15—C16	1.417 (6)
O9—N4	1.260 (5)	C17—C18	1.351 (9)
O10—N4	1.219 (5)	C17—H17	0.9300
N1—C9	1.327 (7)	C18—C19	1.410 (8)
N1—C6	1.362 (7)	C18—H18	0.9300
N1—H1	0.859 (10)	C19—C20	1.492 (8)
N2—C19	1.333 (6)	C20—H20A	0.9600
N2—C16	1.362 (6)	C20—H20B	0.9600
N2—H2	0.861 (10)	C20—H20C	0.9600
C1—C2	1.389 (7)	C21—C22	1.498 (8)
C1—C6	1.426 (7)	C22—H22A	0.9600
C2—C3	1.408 (8)	C22—H22B	0.9600
C2—H2A	0.9300	C22—H22C	0.9600
			
O1—La1—O2	144.47 (11)	O5'—N3'—O6'	119.9 (7)
O1—La1—O6'	75.4 (9)	O7'—N3'—La1	176.0 (13)
O2—La1—O6'	106.1 (9)	O5'—N3'—La1	63.9 (12)
O1—La1—O2^i^	84.12 (11)	O6'—N3'—La1	56.0 (12)
O2—La1—O2^i^	67.27 (11)	O10—N4—O8	121.1 (5)
O6'—La1—O2^i^	129.1 (6)	O10—N4—O9	121.9 (5)
O1—La1—O3	75.49 (11)	O8—N4—O9	117.0 (4)
O2—La1—O3	116.55 (11)	O10—N4—La1	178.6 (4)
O6'—La1—O3	136.3 (9)	O8—N4—La1	60.0 (2)
O2^i^—La1—O3	78.84 (11)	O9—N4—La1	57.0 (2)
O1—La1—O4	125.74 (11)	O1—C1—C2	124.7 (5)
O2—La1—O4	75.19 (11)	O1—C1—C6	119.3 (4)
O6'—La1—O4	142.2 (6)	C2—C1—C6	115.9 (4)
O2^i^—La1—O4	87.10 (11)	C1—C2—C3	121.3 (5)
O3—La1—O4	50.30 (11)	C1—C2—H2A	119.4
O1—La1—O9	87.53 (12)	C3—C2—H2A	119.4
O2—La1—O9	126.22 (10)	C4—C3—C2	122.1 (6)
O6'—La1—O9	66.1 (6)	C4—C3—H3	118.9
O2^i^—La1—O9	159.37 (12)	C2—C3—H3	118.9
O3—La1—O9	80.82 (13)	C3—C4—C5	119.9 (5)
O4—La1—O9	82.54 (14)	C3—C4—H4	120.1
O1—La1—O5'	82.3 (8)	C5—C4—H4	120.1
O2—La1—O5'	73.8 (6)	C7—C5—C4	124.9 (5)
O6'—La1—O5'	49.1 (5)	C7—C5—C6	117.3 (6)
O2^i^—La1—O5'	82.6 (7)	C4—C5—C6	117.9 (5)
O3—La1—O5'	152.3 (8)	N1—C6—C5	118.7 (5)
O4—La1—O5'	148.9 (7)	N1—C6—C1	118.4 (4)
O9—La1—O5'	115.0 (6)	C5—C6—C1	123.0 (5)
O1—La1—O6	87.0 (3)	C8—C7—C5	121.2 (5)
O2—La1—O6	91.4 (3)	C8—C7—H7	119.4
O6'—La1—O6	14.7 (7)	C5—C7—H7	119.4
O2^i^—La1—O6	123.18 (19)	C7—C8—C9	121.0 (5)
O3—La1—O6	150.6 (3)	C7—C8—H8	119.5
O4—La1—O6	139.4 (2)	C9—C8—H8	119.5
O9—La1—O6	75.0 (2)	N1—C9—C8	117.4 (6)
O5'—La1—O6	40.6 (6)	N1—C9—C10	118.7 (5)
O1—La1—O5	75.8 (2)	C8—C9—C10	123.9 (5)
O2—La1—O5	77.14 (18)	C9—C10—H10A	109.5
O6'—La1—O5	54.1 (6)	C9—C10—H10B	109.5
O2^i^—La1—O5	75.98 (17)	H10A—C10—H10B	109.5
O3—La1—O5	143.3 (2)	C9—C10—H10C	109.5
O4—La1—O5	151.49 (17)	H10A—C10—H10C	109.5
O9—La1—O5	120.07 (17)	H10B—C10—H10C	109.5
O5'—La1—O5	9.0 (7)	O2—C11—C12	125.4 (4)
O6—La1—O5	47.51 (16)	O2—C11—C16	117.6 (4)
O1—La1—O8	133.29 (12)	C12—C11—C16	117.0 (4)
O2—La1—O8	78.27 (10)	C11—C12—C13	121.1 (5)
O6'—La1—O8	73.6 (7)	C11—C12—H12	119.5
O2^i^—La1—O8	142.52 (10)	C13—C12—H12	119.5
O3—La1—O8	105.23 (13)	C14—C13—C12	123.0 (5)
O4—La1—O8	69.72 (12)	C14—C13—H13	118.5
O9—La1—O8	48.04 (11)	C12—C13—H13	118.5
O5'—La1—O8	102.1 (8)	C13—C14—C15	118.5 (5)
O6—La1—O8	70.12 (18)	C13—C14—H14	120.8
O5—La1—O8	111.0 (2)	C15—C14—H14	120.8
O1—La1—C21	100.66 (13)	C14—C15—C17	124.4 (5)
O2—La1—C21	94.73 (12)	C14—C15—C16	119.0 (5)
O6'—La1—C21	149.1 (7)	C17—C15—C16	116.6 (5)
O2^i^—La1—C21	79.82 (12)	N2—C16—C15	119.6 (4)
O3—La1—C21	25.22 (12)	N2—C16—C11	119.1 (4)
O4—La1—C21	25.30 (12)	C15—C16—C11	121.4 (4)
O9—La1—C21	83.26 (15)	C18—C17—C15	121.0 (5)
O5'—La1—C21	161.7 (6)	C18—C17—H17	119.5
O6—La1—C21	156.65 (18)	C15—C17—H17	119.5
O5—La1—C21	155.77 (18)	C17—C18—C19	121.3 (5)
O8—La1—C21	89.09 (13)	C17—C18—H18	119.4
C1—O1—La1	168.0 (3)	C19—C18—H18	119.4
C11—O2—La1	125.3 (2)	N2—C19—C18	117.3 (5)
C11—O2—La1^i^	121.7 (2)	N2—C19—C20	118.3 (5)
La1—O2—La1^i^	112.73 (10)	C18—C19—C20	124.3 (5)
C21—O3—La1	94.4 (3)	C19—C20—H20A	109.5
C21—O4—La1	93.3 (3)	C19—C20—H20B	109.5
N3—O5—La1	96.5 (4)	H20A—C20—H20B	109.5
N3'—O5'—La1	91.4 (12)	C19—C20—H20C	109.5
N3—O6—La1	97.6 (4)	H20A—C20—H20C	109.5
N3'—O6'—La1	99.6 (13)	H20B—C20—H20C	109.5
N4—O8—La1	95.9 (3)	O3—C21—O4	120.8 (5)
N4—O9—La1	99.0 (3)	O3—C21—C22	119.3 (5)
C9—N1—C6	124.4 (4)	O4—C21—C22	119.8 (5)
C9—N1—H1	116 (4)	O3—C21—La1	60.3 (3)
C6—N1—H1	119 (4)	O4—C21—La1	61.4 (3)
C19—N2—C16	124.2 (4)	C22—C21—La1	168.3 (4)
C19—N2—H2	114 (3)	C21—C22—H22A	109.5
C16—N2—H2	122 (3)	C21—C22—H22B	109.5
O7—N3—O5	121.7 (5)	H22A—C22—H22B	109.5
O7—N3—O6	120.1 (5)	C21—C22—H22C	109.5
O5—N3—O6	118.1 (5)	H22A—C22—H22C	109.5
O7'—N3'—O5'	120.2 (7)	H22B—C22—H22C	109.5
O7'—N3'—O6'	120.0 (7)		
			
O2—La1—O1—C1	29.7 (17)	La1—O6—N3—O5	−4.4 (5)
O6'—La1—O1—C1	127.1 (18)	La1—O5'—N3'—O7'	−179.9 (17)
O2^i^—La1—O1—C1	−5.9 (16)	La1—O5'—N3'—O6'	0.1 (17)
O3—La1—O1—C1	−85.8 (16)	La1—O6'—N3'—O7'	179.9 (19)
O4—La1—O1—C1	−88.0 (16)	La1—O6'—N3'—O5'	−0.1 (19)
O9—La1—O1—C1	−167.0 (16)	O1—La1—N3'—O5'	−98.4 (18)
O5'—La1—O1—C1	77.4 (17)	O2—La1—N3'—O5'	48.0 (18)
O6—La1—O1—C1	117.9 (16)	O6'—La1—N3'—O5'	179.9 (18)
O5—La1—O1—C1	71.1 (16)	O2^i^—La1—N3'—O5'	−18.3 (19)
O8—La1—O1—C1	176.7 (16)	O3—La1—N3'—O5'	−115 (2)
C21—La1—O1—C1	−84.3 (16)	O4—La1—N3'—O5'	101.2 (18)
O1—La1—O2—C11	135.0 (3)	O9—La1—N3'—O5'	174.2 (18)
O6'—La1—O2—C11	47.8 (7)	O6—La1—N3'—O5'	142 (2)
O2^i^—La1—O2—C11	174.0 (4)	O5—La1—N3'—O5'	−15.6 (18)
O3—La1—O2—C11	−122.4 (3)	O8—La1—N3'—O5'	126.3 (18)
O4—La1—O2—C11	−92.9 (3)	O1—La1—N3'—O6'	81.7 (19)
O9—La1—O2—C11	−24.1 (4)	O4—La1—N3'—O6'	−78.7 (19)
O5'—La1—O2—C11	85.4 (8)	O5'—La1—N3'—O6'	−179.9 (18)
O6—La1—O2—C11	48.3 (3)	O5—La1—N3'—O6'	164.5 (19)
O5—La1—O2—C11	94.0 (4)	La1—O8—N4—O10	179.0 (5)
O8—La1—O2—C11	−21.1 (3)	La1—O8—N4—O9	0.9 (5)
C21—La1—O2—C11	−109.2 (3)	La1—O9—N4—O10	−179.0 (5)
O1—La1—O2—La1^i^	−38.9 (2)	La1—O9—N4—O8	−1.0 (5)
O6'—La1—O2—La1^i^	−126.2 (7)	O1—La1—N4—O8	−163.4 (3)
O2^i^—La1—O2—La1^i^	0.0	O2—La1—N4—O8	2.7 (3)
O3—La1—O2—La1^i^	63.64 (15)	O6'—La1—N4—O8	−99.8 (11)
O4—La1—O2—La1^i^	93.10 (13)	O2^i^—La1—N4—O8	56.4 (5)
O9—La1—O2—La1^i^	161.98 (14)	O3—La1—N4—O8	120.8 (3)
O5'—La1—O2—La1^i^	−88.6 (8)	O4—La1—N4—O8	73.5 (3)
O6—La1—O2—La1^i^	−125.68 (16)	O9—La1—N4—O8	179.0 (5)
O5—La1—O2—La1^i^	−80.0 (2)	O5'—La1—N4—O8	−74.3 (8)
O8—La1—O2—La1^i^	164.96 (15)	O6—La1—N4—O8	−84.4 (5)
C21—La1—O2—La1^i^	76.88 (14)	O5—La1—N4—O8	−79.1 (4)
O1—La1—O3—C21	176.5 (3)	C21—La1—N4—O8	96.7 (3)
O2—La1—O3—C21	32.4 (3)	O1—La1—N4—O9	17.6 (4)
O6'—La1—O3—C21	−133.9 (10)	O2—La1—N4—O9	−176.3 (3)
O2^i^—La1—O3—C21	89.8 (3)	O6'—La1—N4—O9	81.2 (11)
O4—La1—O3—C21	−5.8 (3)	O2^i^—La1—N4—O9	−122.6 (4)
O9—La1—O3—C21	−93.7 (3)	O3—La1—N4—O9	−58.2 (3)
O5'—La1—O3—C21	138.6 (12)	O4—La1—N4—O9	−105.5 (3)
O6—La1—O3—C21	−128.3 (4)	O5'—La1—N4—O9	106.7 (8)
O5—La1—O3—C21	137.1 (3)	O6—La1—N4—O9	96.6 (5)
O8—La1—O3—C21	−51.9 (3)	O5—La1—N4—O9	101.9 (4)
O1—La1—O4—C21	8.5 (4)	O8—La1—N4—O9	−179.0 (5)
O2—La1—O4—C21	−139.4 (3)	C21—La1—N4—O9	−82.3 (3)
O6'—La1—O4—C21	123.4 (15)	La1—O1—C1—C2	−94.4 (16)
O2^i^—La1—O4—C21	−72.1 (3)	La1—O1—C1—C6	85.9 (16)
O3—La1—O4—C21	5.8 (3)	O1—C1—C2—C3	179.6 (5)
O9—La1—O4—C21	90.0 (3)	C6—C1—C2—C3	−0.7 (7)
O5'—La1—O4—C21	−142.6 (15)	C1—C2—C3—C4	0.9 (9)
O6—La1—O4—C21	146.3 (5)	C2—C3—C4—C5	−0.9 (9)
O5—La1—O4—C21	−125.1 (5)	C3—C4—C5—C7	178.6 (6)
O8—La1—O4—C21	137.9 (3)	C3—C4—C5—C6	0.6 (8)
O1—La1—O5—N3	96.7 (6)	C9—N1—C6—C5	−1.3 (7)
O2—La1—O5—N3	−106.5 (6)	C9—N1—C6—C1	178.5 (4)
O6'—La1—O5—N3	14.5 (10)	C7—C5—C6—N1	1.1 (7)
O2^i^—La1—O5—N3	−176.0 (6)	C4—C5—C6—N1	179.3 (5)
O3—La1—O5—N3	136.0 (5)	C7—C5—C6—C1	−178.6 (5)
O4—La1—O5—N3	−120.6 (4)	C4—C5—C6—C1	−0.4 (7)
O9—La1—O5—N3	18.1 (7)	O1—C1—C6—N1	0.4 (6)
O5'—La1—O5—N3	−39 (4)	C2—C1—C6—N1	−179.3 (4)
O6—La1—O5—N3	−2.5 (3)	O1—C1—C6—C5	−179.8 (4)
O8—La1—O5—N3	−34.7 (6)	C2—C1—C6—C5	0.5 (7)
C21—La1—O5—N3	−179.0 (4)	C4—C5—C7—C8	−179.0 (6)
O1—La1—O5'—N3'	77.3 (16)	C6—C5—C7—C8	−1.0 (8)
O2—La1—O5'—N3'	−129.3 (18)	C5—C7—C8—C9	1.0 (9)
O6'—La1—O5'—N3'	−0.1 (10)	C6—N1—C9—C8	1.2 (7)
O2^i^—La1—O5'—N3'	162.3 (18)	C6—N1—C9—C10	−179.8 (5)
O3—La1—O5'—N3'	114.1 (18)	C7—C8—C9—N1	−1.0 (8)
O4—La1—O5'—N3'	−126.1 (12)	C7—C8—C9—C10	180.0 (6)
O9—La1—O5'—N3'	−6(2)	La1—O2—C11—C12	−84.2 (5)
O6—La1—O5'—N3'	−17.1 (10)	La1^i^—O2—C11—C12	89.2 (5)
O5—La1—O5'—N3'	120 (5)	La1—O2—C11—C16	97.3 (4)
O8—La1—O5'—N3'	−55.5 (17)	La1^i^—O2—C11—C16	−89.2 (4)
C21—La1—O5'—N3'	178.0 (13)	O2—C11—C12—C13	−176.7 (4)
O1—La1—O6—N3	−71.0 (6)	C16—C11—C12—C13	1.8 (7)
O2—La1—O6—N3	73.5 (6)	C11—C12—C13—C14	0.9 (8)
O6'—La1—O6—N3	−108 (3)	C12—C13—C14—C15	−2.1 (8)
O2^i^—La1—O6—N3	10.0 (8)	C13—C14—C15—C17	−179.9 (5)
O3—La1—O6—N3	−123.7 (4)	C13—C14—C15—C16	0.6 (7)
O4—La1—O6—N3	142.1 (3)	C19—N2—C16—C15	−2.2 (7)
O9—La1—O6—N3	−159.2 (6)	C19—N2—C16—C11	176.3 (4)
O5'—La1—O6—N3	10.7 (11)	C14—C15—C16—N2	−179.4 (4)
O5—La1—O6—N3	2.5 (3)	C17—C15—C16—N2	1.0 (6)
O8—La1—O6—N3	150.5 (7)	C14—C15—C16—C11	2.1 (6)
C21—La1—O6—N3	178.9 (4)	C17—C15—C16—C11	−177.5 (4)
O1—La1—O6'—N3'	−92.4 (17)	O2—C11—C16—N2	−3.2 (6)
O2—La1—O6'—N3'	50.8 (19)	C12—C11—C16—N2	178.2 (4)
O2^i^—La1—O6'—N3'	−23 (2)	O2—C11—C16—C15	175.3 (4)
O3—La1—O6'—N3'	−142.0 (14)	C12—C11—C16—C15	−3.3 (6)
O4—La1—O6'—N3'	137.2 (11)	C14—C15—C17—C18	−179.3 (5)
O9—La1—O6'—N3'	174 (2)	C16—C15—C17—C18	0.3 (7)
O5'—La1—O6'—N3'	0.1 (10)	C15—C17—C18—C19	−0.5 (9)
O6—La1—O6'—N3'	49 (2)	C16—N2—C19—C18	2.0 (7)
O5—La1—O6'—N3'	−9.5 (12)	C16—N2—C19—C20	−177.6 (4)
O8—La1—O6'—N3'	123 (2)	C17—C18—C19—N2	−0.5 (8)
C21—La1—O6'—N3'	−178.7 (9)	C17—C18—C19—C20	179.0 (5)
O1—La1—O8—N4	21.5 (4)	La1—O3—C21—O4	10.7 (5)
O2—La1—O8—N4	−177.3 (3)	La1—O3—C21—C22	−166.5 (5)
O6'—La1—O8—N4	71.8 (9)	La1—O4—C21—O3	−10.6 (5)
O2^i^—La1—O8—N4	−154.2 (3)	La1—O4—C21—C22	166.6 (5)
O3—La1—O8—N4	−62.7 (3)	O1—La1—C21—O3	−3.4 (3)
O4—La1—O8—N4	−98.9 (3)	O2—La1—C21—O3	−151.3 (3)
O9—La1—O8—N4	−0.5 (3)	O6'—La1—C21—O3	75.9 (18)
O5'—La1—O8—N4	112.3 (7)	O2^i^—La1—C21—O3	−85.4 (3)
O6—La1—O8—N4	86.9 (5)	O4—La1—C21—O3	169.6 (5)
O5—La1—O8—N4	111.6 (3)	O9—La1—C21—O3	82.8 (3)
C21—La1—O8—N4	−82.3 (3)	O5'—La1—C21—O3	−101 (3)
O1—La1—O9—N4	−163.5 (3)	O6—La1—C21—O3	104.1 (9)
O2—La1—O9—N4	4.5 (4)	O5—La1—C21—O3	−82.4 (6)
O6'—La1—O9—N4	−88.4 (11)	O8—La1—C21—O3	130.6 (3)
O2^i^—La1—O9—N4	130.4 (3)	O1—La1—C21—O4	−173.0 (3)
O3—La1—O9—N4	120.8 (3)	O2—La1—C21—O4	39.2 (3)
O4—La1—O9—N4	69.9 (3)	O6'—La1—C21—O4	−93.6 (18)
O5'—La1—O9—N4	−83.2 (9)	O2^i^—La1—C21—O4	105.0 (3)
O6—La1—O9—N4	−76.0 (4)	O3—La1—C21—O4	−169.6 (5)
O5—La1—O9—N4	−91.6 (4)	O9—La1—C21—O4	−86.8 (3)
O8—La1—O9—N4	0.6 (3)	O5'—La1—C21—O4	89 (3)
C21—La1—O9—N4	95.4 (3)	O6—La1—C21—O4	−65.5 (9)
La1—O5—N3—O7	−175.8 (4)	O5—La1—C21—O4	108.1 (6)
La1—O5—N3—O6	4.4 (5)	O8—La1—C21—O4	−39.0 (3)
La1—O6—N3—O7	175.7 (4)		

Symmetry codes: (i) −*x*+1, −*y*+1, −*z*+1.

### Hydrogen-bond geometry (Å, °)

**Table:**

*D*—H···*A*	*D*—H	H···*A*	*D*···*A*	*D*—H···*A*
N1—H1···O3	0.86 (1)	2.06 (1)	2.910 (5)	173 (5)
N2—H2···O4	0.86 (1)	2.20 (3)	2.926 (5)	143 (5)
